# Psychometric Properties of the Abdominal Pain Index (API) in the Iranian Adolescent Population

**DOI:** 10.1155/2020/2632139

**Published:** 2020-12-30

**Authors:** Sepideh Hoseini, Mahdi Jafari, Zahra Asl Soleimani, Kaveh Qaderi Bagajan, Meysam Sadeghi, Shadi Zolfaghari

**Affiliations:** ^1^Department of Clinical Psychology, School of Medicine, Shahid Beheshti University of Medical Sciences, Tehran, Iran; ^2^Department of Clinical Psychology, Student Research Committee, University of Social Welfare and Rehabilitation Sciences, Tehran, Iran; ^3^Department of Clinical Psychology, School of Psychology and Education Sciences, Allameh Tabataba'i University, Tehran, Iran; ^4^Institute for Cognitive Sciences Studies Cognitive Psychology Department, Tehran, Iran; ^5^Allameh Tabataba'i University, Tehran, Iran

## Abstract

Considering the high prevalence of abdominal pain in children and adolescents in Iran, it is essential to use appropriate screening tools. One of the most comprehensive, yet concise, tools for this purpose is the Abdominal Pain Index (API). This study aimed to investigate the psychometric properties of the Persian version of the self-report API in adolescents. In this descriptive study, A total of 162 Iranian adolescents in the age range of 12 to 18 years were considered as the sample group, which included two groups of school students (*n* = 125) and adolescent patients with abdominal pain (*n* = 37). Clinical sample was selected by the available sampling method, and nonclinical sample was selected by the cluster sampling method. Adolescents in the sample group were selected from both clinical and nonclinical groups in order to evaluate differential validity. Instruments, including API, somatic symptoms subscale of the General Health Questionnaire (GHQ), and McGill Pain Questionnaire (MPQ), were also completed for the participants. Also, to evaluate the construct validity of API, exploratory and confirmatory factor analysis methods were applied. The exploratory factor analysis identified one general factor, and the confirmatory factor analysis results show the model's satisfactory fitting. Also, the researchers' hypothesis, i.e., API is a single-factor model (with five items), was approved. The reliability coefficient of the questionnaire was satisfactory for the total scale (*α* < 0.7). This study showed that API could be used with considerable confidence for Iranian children and adolescents with chronic pain.

## 1. Introduction

Chronic pain refers to persistent and recurrent pain [1], which lasts longer than three months [2]. This disorder has a predominantly adolescent-onset and affects approximately 11–38% of the youth [3]. It is a relatively common disorder, with a prevalence of 24% in Iran in 2015 [4]. Patients with chronic pain may experience cognitive problems and find it difficult to carry out their normal activities of daily living [5, 6]. Generally, biological, psychological, and social factors are involved in the development of this disorder. However, in patients with no pathological cause for their disease, psychological factors play a major role [7, 8].

Chronic pain has many forms, including back pain, headache, muscle pain, and postsurgical pain. One of the chronic pain disorders, which is most commonly seen in children and adolescents, is abdominal pain, occurring in 5–8% of patients referred to the emergency department [9]. One of the major challenges of physicians is the diagnosis of this disorder [10], as only a few patients require emergency care, and nearly 20–30% have nonspecific abdominal pain [11]. This disorder has a wide range of symptoms, including fatigue, sleep and mood disorders, reduced performance, academic challenges, school attendance problems, and decreased quality of life [7, 12].

Pain can be assessed in three ways, including self-report scales, behavioral tools, and physiological measures [13]. Self-report scales are considered the gold standard and are the most valid method to measure pain [14]. Behavioral tools measure pain by assessing its behavioral manifestations such as crying, facial expressions, posture, and body movements. These tools are especially useful in cases where self-report scales are not applicable, such as infants or patients with severe cognitive or communication disorders [15, 16]. Physiological measures assess pain through pain-related physiological changes such as heart rate, blood pressure, and respiration rate [17].

Since pain is a personal experience and the gold standard to measuring it is self-report tools [14], several scales have been developed for this purpose. Some of these scales to measuring pain in children and adolescents include Adolescent Pediatric Pain Tool (APPT) [18], Faces Pain Scale (FPS) [19], Faces Pain Rating Scale (FPRS) [20], Oucher Scale (OS) [21], and Abdominal Pain Index (API). All of these scales measure pain in youth, but the APPT, FPS, FPRS, and OS measure the intensity of acute pain, whereas the API is designed to measure recurrent or chronic abdominal pain in youth [22]. Specifically, the API has five items that assess the frequency, duration, and severity of pain episodes.

Considering the high prevalence of abdominal pain in children and adolescents in Iran, it is essential to use appropriate screening tools. One of the most comprehensive, yet concise, tools for the evaluation of abdominal pain is API. However, the use of this questionnaire in the Iranian population requires the validation of its Persian version. Therefore, the present study aimed to investigate the psychometric properties of the Persian version of the self-report API in adolescents due to a lack of similar research in Iran. For this purpose, the construct validity was evaluated by exploratory and confirmatory factor analysis and also differential and convergent validity methods. Also, reliability was measured through test-retest and Cronbach's alpha methods. Given the single-factor structure of the API [22], it was hypothesized that the Persian version of the API in Iranian adolescents would also have only one factor.

## 2. Methods

In terms of data collection and methodology, a research study with a descriptive design is considered. Correlational analysis, goodness of fit indices, factor analysis, and reliability analysis were used to study the correlation of variables in the questionnaires and to examine the theoretical framework and standardization of the questionnaires. In addition to factor analysis, convergent and divergent validity methods were used to assess the validity of the questionnaire. Somatic symptoms subscale of the General Health Questionnaire (GHQ) and McGill Pain Questionnaire (MPQ) were used to assess the convergent validity of the questionnaire.

Although these instruments are not designed for adolescents, they were selected for several reasons: First, most of the research in Iran on pain-related problems in adolescents used Made Questionnaire [23, 24] in which this could reduce the validity and reliability of the instrument due to its low research background. Also, adolescent pain questionnaires such as Bath Adolescent Pain Questionnaire (BAPQ) [25] have not been validated in Iran. And finally, somatic symptoms on the General Health Questionnaire and the McGill Pain Questionnaire have been used in other studies related to the pain of Iranian adolescents [26–28].

### 2.1. Study Population and Sampling Method

Since the present study aimed to validate the five-item API based on the factor analysis, the minimum sample size was considered to be 75 people. Considering the possible sample attrition, finally, 162 Iranian adolescents in the age range of 12 to 18 years were considered as the sample group, of which 37 adolescents had abdominal pain and another 125 adolescents did not have it. Adolescents in the sample group were selected from both clinical and nonclinical groups in order to evaluate divergent validity. The nonclinical subjects were selected via cluster sampling. For this purpose, five districts of Tehran were selected by lot among different districts of the city to select the nonclinical group, and six schools were selected among the schools of each district. Next, two classes were selected by lot from each school, and sampling was carried out. On the other hand, the clinical group was selected among patients, referred to the Pediatric Medical Center and two private clinics. The nonclinical and clinical groups included adolescents aged 12–18 years.

The inclusion criteria for the nonclinical group were as follows:

1. No abdominal pain in the past month
2. Lack of nonabdominal pain for more than two days in the past week
3. No experience of chronic pain

On the other hand, the inclusion criteria for the clinical group were as follows:

1. Abdominal pain at least three times a month
2. No history of chronic disease
3. No organic diseases diagnosed by the physician

### 2.2. Research Tools

#### 2.2.1. API

This scale has two forms of self-report and parental reporting and includes five items and examines the number of days with pain, number of pain episodes per day, typical pain episode duration, typical pain intensity, and the most severe period of pain over the past two weeks [22]. The frequency of pain is scored on a six-point scale, ranging from “none” (score 0) to “every day” (score 5). The usual frequency of pain is also scored on a six-point scale (never, once a day, 2-3 times a day, 4-5 times a day, ≥6 times a day, and persistent pain throughout the day). Also, pain duration is assessed on a nine-point scale (never, a few minutes, about half an hour per day, 1-2 hours per day, 3-4 hours per day, 5-6 hours per day, most of the day, and all day). Finally, the most common and severe pain intensity is calculated on an 11-point scale, ranging from “no pain” (score 0) to “maximum pain” (score 10). The concurrent validity, discriminant, construct validity, and high internal consistency of the scale were confirmed [1].

API was first translated into Persian. In the first step, two translators fluent in English translated the questionnaire into Persian, and then two other professional translators back-translated it into English. The original version and the translated version were then compared by a Persian-speaking person familiar with the English language and subjects' terminology, and the necessary revisions were made. In the pilot study, the questionnaire was administered to 20 adolescents. Our purpose was to determine whether adolescents consider different items of the questionnaire to be in accordance with the purpose of the test administrator and to determine if an item has a single impression among adolescents; this was achieved by talking to the participants and examining their understanding of each item. Finally, the necessary revisions were made.

#### 2.2.2. Somatic Symptoms Subscale of GHQ

GHQ [29] is one of the common self-administered questionnaires with various dimensions for assessing nonpsychotic disorders. It is not used for diagnostic purposes, but initial screening. It contains 28 questions, the first seven of which belong to the somatic symptoms subscale. This subscale examines the physical symptoms that a person has experienced in the past month. All items of GHQ have four options, scored on a Likert scale. Each of the four-option questions is scored from 0 to 3; therefore, the score of each person varies from 0 to 21 for each subscale. Several studies have examined the psychometric properties of this questionnaire, all of which have shown its favorable psychometric properties [30–32].

#### 2.2.3. Short-Form MPQ-2 (SF-MPQ-2)

This questionnaire, which was first developed by Melzac [33], contains three main domains (sensory, affective, and evaluative) and is completed by the patient to describe the characteristics of his/her pain experience. This questionnaire includes a pain severity scale and quantifies clinical pain. Its short form contains 15 questions, scored on a four-point Likert scale, ranging from “no pain” (score 0) to “severe pain” (score 3). Overall, three scores are calculated for the sensory, affective, and evaluative domains.

Various studies have examined the psychometric properties of MPQ [34–36] and reported its high reliability and validity. In Iran, the reliability and validity of the short version of this questionnaire were assessed by Vakil Zadeh and Nakhaee [37]. Also, internal consistency was examined using exploratory and confirmatory factor analyses and confirmed by Cronbach's alpha and item-scale correlation coefficient. Generally, lower scores of this questionnaire indicate less severe symptoms. The first 11 items are related to the sensory domain, and the next four items are related to the emotional domain.

## 3. Results

The analysis of demographic data showed that 55.3% of the study samples were 12–14 years old, 30.2% were 15-16 years old, and 14.5% were 17-18 years old. Also, 74.3% of the study samples were girls, while 25.7% were boys. The mean and standard deviation of the sample age were 14.33 and 1.63, respectively.

In order to evaluate the construct validity of the questionnaire, exploratory and confirmatory factor analysis methods were used. At first, the Kaiser–Meyer–Olkin (KMO) test was carried out to assess the five-item API for factor analysis; the measured coefficient was found to be satisfactory (KMO = 0.78). Also, the result of Bartlett's test of sphericity was significant at *P* < 0.0001 (*χ*^2^ = 307.82).  Table 1 presents the information related to the extracted factors.

The present study identified one factor, which could explain 67.84% of variance in abdominal pain. The factor loadings of five questions on one general factor (or more) are presented in Table 2.

Considering the correlation coefficient of 0.30 as the minimum acceptable factor loading of each item on the extracted factor, the factor loadings of five items on one factor are reported in Table 2.

**Figure 1:**
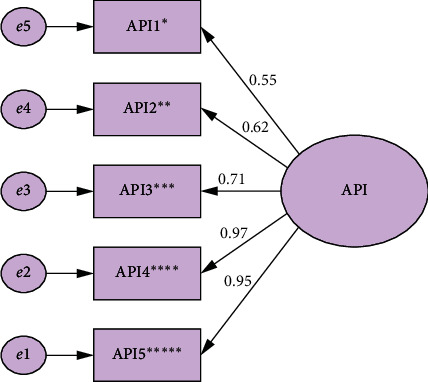
First-order confirmatory factor analysis with standardized coefficients. ^*∗*^Frequency. ^∗∗^Frequency per day. ^∗∗∗^Duration. ^∗∗∗∗^Severity. ^∗∗∗∗∗^Most severity.

As seen in Figure 1, in order to investigate the construct validity of the questionnaire, the first-order confirmatory factor analysis was performed. AMOS software was used for the confirmatory factor analysis.

Based on the results presented in Table 3, it can be concluded that the model is well-suited for explaining abdominal pain. Also, the researchers' hypothesis, i.e., API is a single-factor model (with five items), was approved. The results of confirmatory factor analysis indicated that no items had a factor loading below 0.4. Moreover, the somatic symptoms subscale of GHQ and MPQ were used to investigate the convergent and divergent validity of API. The correlation coefficients of the variables are reported in Table 4.

The present results are in line with previous studies, which indicated a positive correlation between abdominal pain and the subscales of somatic symptoms and pain intensity (*P* < 0.01). Therefore, analysis of convergent validity confirmed the validity of API. An independent *t*-test was also used to evaluate the differential validity of API variables in nonclinical and clinical groups. The results showed that the mean abdominal pain of patients (18/02) was significantly higher than that of nonclinical subjects (3/83), which indicates the good differential validity of the questionnaire (*P* < 0.001).

Also, to assess the reliability of API, Cronbach's alpha and test-retest methods were applied. Cronbach's alpha obtained in this measure (0/862) showed that the reliability coefficient is satisfactory for the total scale (*α* < 0.7).

## 4. Discussion and Conclusion

As mentioned earlier “the API was developed to characterize the pain experience of youth with recurrent or chronic abdominal pain” [22], Walker and colleagues attempted to develop a five-item questionnaire (API) as a comprehensive scale for assessing the adolescents' abdominal pain and determining the extent of pain perception in clinical and nonclinical situations. Researchers have reported the favorable psychometric properties and diagnostic power of this scale and introduced it as a valid tool. Therefore, it is used widely for different groups in different countries with different cultures and languages.

The purpose of this study was to assess the validity and reliability of the Abdominal Pain Index (API) in the Iranian adolescents. The results showed that the abdominal pain questionnaire for Iranian adolescents with chronic pain has a single-factor structure. The results of the present study also showed that API has good reliability, based on the internal consistency coefficient and test-retest results; this finding is consistent with the results of previous studies (e.g., [1]). Also consistent with [1], discriminant validity was supported by the significantly higher API scores observed among the clinical group compared with the nonclinical group.

A review of the literature showed that abdominal pain was significantly correlated with a range of symptoms, including fatigue, sleep and mood disorders, reduced performance, academic challenges, school attendance problems, and decreased quality of life [12]. Accordingly, in the present study, the subscales of somatic symptoms and pain severity were considered to evaluate the convergent validity of this questionnaire. Significant positive correlations were observed between API and the subscales of pain severity and somatic symptoms indicating the convergent validity of API. Consistent with this result, Walker et al. [22] found that there was a significant relationship between API and anxiety, fear of pain, and pain intensity.

Since different ethnicities, races, and social groups were sampled in five districts of Tehran in the present study, it is possible to generalize the results to Farsi speakers. However, the impact of ethnicity and culture was not examined in this study; therefore, caution should be taken in generalizing the results to specific cultural, racial, and dialect groups. Also, the results of the present study were obtained using questionnaires and self-assessments. Therefore, variables, such as responsiveness, external motives, and psychological status at the time of data collection, may have potential effects on their response. It is recommended that future studies use more comprehensive and objective methods, such as direct observation of the patient's behaviors.

According to the available literature, accurate assessment of pain perception plays an important role in the assessment and treatment of chronic pain. Therefore, it is clinically important to have a comprehensive scale, with favorable psychometric properties, to provide accurate assessments of the chronic pain. The results of the present study confirmed the validity and reliability of API in Iranian adolescents; therefore, this scale can meet the urgent need to evaluate chronic pain. Overall, the present results showed that API could be used with considerable confidence in Iranian children and adolescents with chronic pain.

## Figures and Tables

**Table 1:** The eigenvalues and percentages of variance explained by the extracted factors of API.

Statistic factor	1
Eigenvalues	39.3
Percentage of explained variance	84.67
Cumulative percentage of total of variance	84.67

**Table 2:** Principal component analysis and factor loadings for five API items.

Items	Factor
	1
API4: severity	0.927
API5: most severity	0.910
API3: duration	0.786
API2: frequency per day	0.764
API1: frequency	0.709

**Table 3:** Confirmatory factor analysis and fit indices.

Fit indices	Amount	Limit
*χ* ^2^/*df*	2.83	<3
RMSEA	0.08	<0.1
CFI	0.96	>0.9
NFI	0.95	>0.9
GFI	0.96	>0.9
AGFI	0.94	>0.9

RMSEA: root mean error of approximation; CFI: comparative fit index; NFI: normed fit index; GFI: goodness of fit index; AGFI: adjusted goodness of fit index.

**Table 4:** Correlation coefficients of API with the somatic symptoms of GHQ and SF-MPQ-2.

Domain/measure	1	2	3
1. API	1	—	—
2. Somatic symptoms subscale of GHQ	0.45^*∗*^	1	—
3. SF-MPQ-2	0.60^*∗*^	0.48^*∗*^	1

^*∗*^ *P* < 0.01.

## Data Availability

The data that support the findings of this study are available from the corresponding author upon reasonable request.
